# Dealing With Rape Cases in Ghana: The Law, the Victim and the Offender

**DOI:** 10.5964/sotrap.12319

**Published:** 2023-12-05

**Authors:** Jacob Mensah Agboli

**Affiliations:** 1 Narcotics Control Commission, Accra, Ghana; Department of Psychiatry and Psychotherapy, University Medical Center Mainz, Mainz, Germany

**Keywords:** rape, carnal knowledge, revictimization, stigmatisation, consent, penetration, Ghana

## Abstract

Rape is a criminal offence in all countries of the world. However, what constitutes rape in the legal sense differs from country to country, with many common law countries sharing some similarities in their definition of rape. This paper conducts a critical review of the crime of rape in Ghana by discussing what constitutes rape under Ghanaian law, its key elements which a prosecution has to prove to succeed on a charge of rape, and the challenges of adjudicating rape cases in Ghana, paying particular attention to the victim. Furthermore, the manuscript offers some useful recommendations on improving current practice. Among the challenges identified as militating against the successful prosecution of rape cases in Ghana are the high poverty level resulting in the lack of money to pay for the costs of forensic medical examination by the victim, stigmatisation and ridicule of rape victims, lack of social and psychological support for the victims of rape, the unduly long period adjudication of rape cases take, the gender-specific definition of rape, and the general inadequate or lack of reformative services for rape prisoners. The paper recommends that the state of Ghana should take steps to tackle these challenges in order to promote justice for rape victims, while effectively dealing with the perpetrators of such crimes. These steps could include, the State covering the costs of forensic medical examination for rape victims through the National Health Insurance Scheme, intensifying public education on rape and its consequences, especially on the victim, and amending the definition of rape to make it gender-neutral instead of gender-specific. This paper will be useful to legal, psychological, and sociological researchers in Ghana and the world over.

## Highlights

- This paper discusses the law on rape in Ghana. It defines what rape is according to the criminal laws of Ghana, the types of rape recognised by Ghanaian criminal law and provides the historical context of the law on rape in Ghana. It also identifies the key elements of rape which the prosecution has to prove in order to succeed, supported by case law.
- The paper found that, the offence of rape in Ghana is gender-specific and age-specific as well. In this regard, the law limits the victims of rape to only females and the perpetrators to only males. Age-wise, rape can only be committed on a female of at least 16 years old. By making rape gender and age-specific, Ghana’s criminal law on rape falls short of good examples from Kenya, South Africa, England, the US and Canada that have all made rape gender-neutral.
- The paper identifies the challenges faced by the state in prosecuting rape cases in Ghana. These challenges range from the inability of rape victims to pay for medical examinations, lack of psychological treatment and support for victims, revictimisation of victims by society through ridicule, stigmatisation, and blaming, among others.
- The paper offered some useful recommendations for reforming Ghana’s law on rape. These include the state paying for the medical examination of rape victims, public education, provision of psychotherapy for both victims of rape and their perpetrators, amendment of the definition of rape in Ghana to make it gender-neutral among others.

## Abstract (plain language summary)

**Background and why was this study done?:**

Ghana is a multi-ethnic, multi-religious, and highly moralistic society. As a predominantly Christian country, Ghana’s criminal laws are Judeo-syncretic in nature, criminalising several immoral and reprehensible conducts. Sexual offences constitute some of those conducts. However, statistics in the last decade and beyond have shown that, reported cases of rape have been on the rise, despite the severe punishment attached to the offence.

**What did the researcher do and find?:**

This paper examines the sociocultural, legal, and psychological context/factors responsible for the rise in rape cases in Ghana. The paper first discusses what rape is according to Ghanaian criminal law, what elements the prosecution has to prove in order to succeed on a charge of rape, and what the meaning of those key elements are, supported by case law. The paper then discusses the challenges of adjudicating rape cases in Ghana and offered some useful recommendations to help improve the adjudication of rape cases in Ghana and reduce, if not eradicate, rape cases in Ghana. Using web-sourced data and the practitioner’s critical analysis/review methodology, the paper revealed that, compared to other countries like South Africa, Kenya, the UK, the US, and Canada, rape in Ghana is gender-specific (limited to female victims), age specific (limited to females of 16 years and above) and excludes other forms of sexual intercourse than penile penetration of the vagina of a woman. Challenges in prosecuting rape cases include the inability of victims to pay for their medical examination, lack of social support for victims, stigmatisation of rape victims, lack of psychological treatment for victims and perpetrators, and the relatively long delays in prosecuting rape cases in court.

**What do these findings mean?:**

The paper recommends that the state take steps to deal with these challenges. These include, the state paying for the cost of medical examination of rape victims, compensation for victims, public education to reduce stigmatisation, and fast-tracking the adjudication of rape cases. It is hoped that, when these recommendations are implemented, Ghana will experience a significant decline in rape cases.

The Ghanaian society is a highly moralistic and religious one and abhors criminal commissions, including sexual offences. Sexual offences constitute some of the most reprehensible forms of criminal offences in the Ghanaian society and the world at large. According to Barroso et al. (2019), sexual violence is one of the most serious criminal behaviours, one of the most prevalent around the globe, and one that significantly negatively influences the victims' personal and social lives. Yet, statistics and research suggest that sexual offences are quite common in low-income and middle-income countries like Ghana as well (Coll et al., 2020; Hardt et al., 2023). As common as sexual offences are in low-and-middle-income countries (LMICs) such as Ghana, there is limited [reported] research available (Hardt et al., 2023).

Ghanaians are greeted with media reports of rape and other forms of sexual violence/assault regularly. Meanwhile, rape is highly underreported in Ghana (Boakye, 2009; Boateng, 2015; Dziwornu, 2021; Ghana News Agency, 2023; Reuters, 2020; United Nations Populations Fund [UNFPA], 2020). According to research, rape was Ghana’s fourth most common crime between 2007 and 2011, rising to the third-highest ranking in 2012 (Boateng, 2015). Regarding serious offences (first degree felonies and treasons such as murder, manslaughter, and robberies) rape was the least reported serious offence in 2013 (Boateng, 2015). According to the 2021 Population and Housing Census results of the Ghana Statistical Service (GSS), Ghana’s population stands at 30.8 million (GSS, 2023). In 2016, there were 497 reported cases of rape, a 10% increase over the 2015 figure of 451, whereas in 2017, the number rose to 514, representing a 3.4% increase over the 2016 figure (Ghana Police Service, 2016, 2017). Similarly, statistics from the Domestic Violence and Victim Support Unit (DOVVSU) of the Ghana Police Service revealed that, 305 women were raped in 2020 (Daily Graphic, 2021) while the figure stood at 504 in 2019 (UNICEF, 2020). It must be emphasised that, these figures represent only reported cases and may not be representative of the true state of affairs regarding rape cases in Ghana. Recent statistics suggest that, reported rape cases have declined (Ghana News Agency, 2023). The fact that rape cases are on the decline could mean that things are under control. On the contrary however, as previously mentioned, rape cases are highly underreported in Ghana and, as research has found out, rape cases are incredibly complicated, with many rape victims preferring to keep quiet about the incident since they feel horrible and ashamed (Pappoe & Ardayfio-Schandorf, 1998; Wrigley-Asante et al., 2016).

Some studies have found that, not only is rape pervasive in Ghana but that, there is a rape culture in Ghana (Allotey, 2019; Brew & Nukpezah, 2022). For example, a study by Brew and Nukpezah (2022) found that rape culture is well ingrained in Ghana, and that people's responses to allegations of rape depend on their preconceived notions about who committed the crime, who was the victim, and the circumstances surrounding the rape. Meanwhile, Allotey (2019) has observed that, despite the overwhelming facts to support rape culture in Ghana, many Ghanaians would prefer to deny its existence since it is so ubiquitous and normalised in the society. It has been argued that, the prevalence of sexual violence in Ghana is a result of the country’s rape culture (UNICEF, 2020).

Sexual offences, for that matter, the criminal offence of rape is not limited to Ghana but pervasive in other cultures as well. Ranganathan et al. (2021) from their meta-analysis of measurement and prevalence of sexual harassment in LMIC observed that, although there was significant variation in prevalence estimates between studies and no worldwide comparability, the prevalence was higher in educational institutions than in workplaces. Walker et al. (2019) further found that, nearly half of those who have experienced child sexual abuse experience further sexual exploitation (revictimisation) in the future, whereas Anderson (2016) also stated that, in nations like the United States and the United Kingdom, rape is a recurrent issue that directly affects a sizable section of the female population. According to self-reports, about one in four US women, today have been raped (CDC, 2022); figures of a comparable magnitude have also been observed in Canada and England (Murphy-Oikonen et al., 2022; Walker et al., 2021). Furthermore, according to population-based prevalence research, between 28% and 37% of men in South Africa admitted ever committing a rape, whereas 12–25% of women reported ever experiencing rape (Abrahams et al., 2020). However, while some of these countries have taken or are taking steps to reform their laws and confront the crime of rape heads-on, Ghana seemed not to be doing much in emulating the pacesetting example of these countries.

This paper focusses on the offence of rape in Ghana, primarily based on the fact that, crime statistics over the years showed that rape is the most reported sexual offence in Ghana. While some few reported studies have examined rape in Ghana, their approach is to either focus on the legal/criminal aspect, the sociological/socio-cultural aspect, or the psychological aspect (Boakye, 2009; Donkor, 2019; Norman et al., 2013). Also missing in these studies is a precise examination of the issue of rape from the perspective of the law or offender. Thus, this paper, adopted an eclectic approach in critically examining the issue of rape in Ghana from three perspectives (legal, sociological/socio-cultural, and psychological), while at the same time identifying challenges with prosecuting rape cases in Ghana. The paper is divided into three parts. The first part discusses the offence of rape and the key elements a prosecution has to prove to succeed on a charge of rape in Ghana. The second part discusses the challenges of adjudicating rape cases in Ghana, whereas, the final part concludes the discussions by offering some useful suggestions for dealing with rape cases in Ghana to ensure/promote true justice to the victim, punish the perpetrator for the crime and protect society from such persons.

## Contextualisation of Rape as a Sexual Offence in Ghana

Sexual Offences in Ghana are provided for under chapter six (6) of the Criminal and Other Offences Act, 1960 (Act 29). This chapter is titled “Sexual Offences” and covers sections 97 to 111 of the Act. Under this chapter, the sexual offences recognised in Ghana are rape, defilement, indecent assault, unnatural carnal knowledge, incest, procuration, and prostitution.

In Ghana, section 98 of the Criminal and Other Offences Act, 1960 (Act 29) defines rape as "the carnal knowledge of a girl of sixteen years or above without her consent" (p.49). Similar to this, according to section 1 of the Sexual Offences Act, 2003, of England and Wales, rape is defined legally as the intentional penetration of the vagina, anus, or mouth of another person without the person’s consent. In explaining section 98 of Act 29, the Supreme Court of Ghana, speaking through Justice Jones V.M. Dotse, in the case of *G/CPL Valentino Gligah & EC/1 Abdulai Aziz Atiso v. The Republic [2010] DLSC4149,* held that “the following are the ingredients of Rape:

1. That someone has had carnal knowledge of the victim…
2. That, the someone is the accused person…
3. That the victim was carnally known against her wish” (p. 4153).

In a similar vein, the Supreme Court of Ghana again, in the case of *Richard Banousin v. The Republic [2014] GHASC 10* ruled that the language used to describe and demonstrate the key elements of the alleged offence must be brutally honest in cases involving crimes like rape, defilement, and in fact any sexual offence. For instance, in a rape case, it is necessary to establish the following:

1. That the victim, [in this case Rashida], was known to the offender sexually.
2. That the accused [in this case, the appellant], is the person who had a sexual relationship with the victim.
3. The victim (Rashida), who did not consent to the sex, was carnally known against her will.
4. The victim is at least 16 years old.

Other common law jurisdictions of the world such as Nigeria and New Zealand have similar definitions for rape (Anifowose, 2020; Cozens, 2015). Therefore, the key terms/words that run through these definitions are carnal knowledge/penetration, lack of consent, and female, among others.

## Determination/Explanation of Key Terms in Rape

The first key term in the definition of rape is carnal knowledge/penetration. In explaining/defining what carnal knowledge is/means under Ghanaian law, section 99 of Act 29 states that

“whenever, upon the trial of any person for an offence punishable under this Code, it is necessary to prove carnal knowledge or unnatural carnal knowledge, the carnal knowledge or unnatural carnal knowledge shall be deemed complete upon proof of the least degree of penetration” (p. 62).

From this provision, therefore, carnal knowledge under Ghanaian law is synonymous with penetration, the least degree of it, being sufficient to prove carnal knowledge. The question then is, what amounts to carnal knowledge? In the Ghanaian case of *G/CPL Valentino Gligah & EC/1 Abdulai Aziz Atiso v. The Republic [2010] DLSC4149*, the Supreme Court attempted to answer this question in the following terms:

“Carnal knowledge is the penetration of a woman’s vagina by a man’s penis. It does not really matter how deep or however little the penis went into the vagina. So long as there was some penetration beyond what is known as brush work, penetration would be deemed to have occurred and carnal knowledge taken to have been completed” (p. 4153).

It follows from this decision of the Supreme Court of Ghana therefore that, where a man is accused of or alleged to have had carnal knowledge of a woman, what the prosecution has to prove as far as carnal knowledge is concerned is the least degree of penetration. In other words, the least degree of penetration of the vagina of a woman by a man, “even brushing”, without the woman’s consent constitutes rape under Ghanaian law. It further means that, any form of penetration other than penile penetration of the vagina of the female will not satisfy this requirement.

The next key element of rape in Ghana is female. *Prima facie*, the definition of rape in Ghana means that, rape is a gender-specific crime/offence. Unlike countries like Canada, the United Kingdom, South Africa (Devakumar, 2021), Finland (European Institute for Gender Equality, 2023), the US and all Australian States (Rumney, 2007) that have taken steps to make rape a gender-neutral offence, Ghana has maintained rape as a gender-specific offence. In the US, it has been the law since 1927 that rape was gender-specific, limited to only male accused persons and female victims. However, as the US Department of Justice Archives noted, “that definition, unchanged since 1927, was outdated and narrow. It only included forcible male penile penetration of a female vagina”. The report continued that, “the new definition therefore is that, rape is “the penetration, no matter how slight, of the vagina or anus with any body part or object, or oral penetration by a sex organ of another person, without the consent of the victim.” (United States Department of Justice Archives, 2012, p. 1). For the first time ever, the new definition includes any gender of victim and perpetrator, not just women being raped by men.” (p. 1). Furthermore, unlike the case of the US where the law also acknowledges that rape using an object can be just as horrific as rape by penile penetration of the vagina, Ghanaian law limits rape solely/strictly to penile penetration of the vagina. Consequently, vaginal penetration by an object or even the finger(s) of the man would amount to indecent assault under section 103 of Act 29 and attracts lesser punishment compared to rape. Rape is a first degree felony, punishable by up to life imprisonment whereas indecent assault is a misdemeanour punishable by 6 months to 3 years term of imprisonment. In addition, other forms of sexual behaviour such as oral or anal sex would also not qualify as rape in Ghana because they are not *per vaginum*. Thus, in simple terms, any form of penetration of a female other than vaginal penetration is not rape in Ghana. Consequently, the Court of Appeal ruled in the case of *Hanson v The Republic [1978] GLR 477* that, if the prosecution fails to adduce evidence of the victim’s sexual intercourse with a man by means of the penis penetrating into the vagina, but by any other means such as fingers, tongue or stick, the action will fail.

In South Africa, the Criminal Law (Sexual Offences and Related Matters) Amendment Act 32 of 2007 (Sexual Offences Amendment Act or SOAA) states that rape occurs when a person forces another to have sexual intercourse with them without their consent. Specifically, the SOAA in section 3 defines 'rape' as “the intentional commission of a sexual act with another person under coercive circumstances.” The SOAA also makes it a crime for a person to force another person to rape someone (South African Government, 2023). Evidently clear from this definition of rape in South Africa is the fact that, the definition is gender-neutral. Thus, under South African law, just like under US Federal laws, both men and women can be victims of rape. Australia also defines rape in a gender-neutral manner as the penetration of the vagina, anus, or mouth without consent (McKeever, 2019). The reverse, however, is true in the case of Ghana.

In Ghana, only females can be raped by males. This is because, by definition, rape is incomplete without penile penetration of the vagina of a female. Since only males possess a penis and only females possess a vagina, rape can only be committed on female victims by male perpetrators (I am aware that this is a simplification of gender and sex characteristics, however, for reasons of clarity I decided to choose this wording as used by the law itself). The presumption/jurisprudence underlying this reasoning is that, it is believed that, a man can only penetrate a woman when he achieves erection. That being the case, then once a man achieves an erection and proceeds to penetrate a woman, he has formed the necessary intention (*mens rea*) required to establish rape. It is argued further in this regard that, without the necessary *mens rea,* even if a woman undresses before the man, he would not achieve an erection, let alone proceed to penetrate the woman. According to Tuffour (2022), due to Ghana's sociocultural setting, it is difficult to recognise claims of men being raped by women as genuine and proper. For this reason, it is important to first determine whether the country is prepared to change these dynamics by making rape a gender-neutral offence.

A thorny issue in Ghana is whether a transgendered person (female) satisfies the element of female in the definition of rape. While this issue is yet to come before the courts, it is the writer’s opinion that, to the extent that that person possesses a vagina that can be penetrated/carnally known, the transgendered person would succeed in a charge of rape against another man. Ghana’s laws have no specific provisions directly relating to LGBTQI+, albeit a Bill is currently before the Parliament of Ghana seeking to criminalise all forms of LGBTQI+.

It is the view of the author that, in making rape a gender-specific offence in Ghana, limiting its commission to only males and the victims to only females, the law is extremely discriminatory, archaic and unfair. This is because, science has established that, sexual arousal, which can be broken down into central, peripheral non-genital, and genital arousal, is the activation of physiological systems that regulate sexual activity in both sexes. Men experience an erection after genital excitation, whereas women have vaginal lubrication and clitoral/vulvar (vestibular bulb) congestion (Graziottin, 2004). Therefore, if both sexes become aroused physiologically when about to have sexual intercourse, why is it not possible for a woman to rape a man as well or for a man to rape another man?

The next key element of rape under Ghanaian law is age. In other words, rape in Ghana is age-specific, to wit, 16 years and above. Where a person (male or female) below 16 years of age has sexual intercourse with another person/man, the perpetrator commits defilement on the under 16-year-old child. Here, it is immaterial whether the female child has consented or not, since a child below 16 years of age is presumed to be incapable of consenting to sexual intercourse in Ghana.

The final element the prosecution has to prove in rape cases is the lack of consent. For the start, it must be noted that, consent cannot be presumed when it comes to sexual intercourse, and for that matter, the commission of rape. Thus, consent must be expressed voluntarily and devoid of the presence of any vitiating factor. In every case of rape, hence, the prosecution must establish by way of evidence that the victim did not consent to having sexual intercourse with the accused person. Under Ghanaian law, section 14 of Act 29 has extensive provisions on what constitutes consent and how to prove consent or its lack thereof. In the case of *Agbemenya v. The State [1964] GLR 663*, the Supreme Court of Ghana ruled that the absence of consent, which is anchored under sections 14 and 42(g) of Act 29, is an essential proof of rape. This means that the prosecution must show the lack of consent or the expressed withdrawal of consent even in cases where it was granted, for the cause of action to succeed in a charge of rape. By consent, the law means that, both parties have voluntarily and mutually agreed to have sex and went ahead to do so. It must be emphasised here that, this consent must be total, exist throughout the act and must be given by a sane adult who fully understands the nature of the act. Thus, where a man procures the consent of a female of 16 years or above by deceit, fraud, misrepresentation or intoxication, that consent is totally void. Therefore, in the English case of *R v. Williams [1923] 1 KB 340*, the defendant was a singing coach. He told one of his pupils that he was performing an act to open her air passages to improve her singing. In fact, he was having sexual intercourse with her. The court ruled that her consent was vitiated by fraud as to the nature and quality of the act. Similar to this, defendant, John Flattery (JF) pretended to be a doctor and surgeon in the case *R v. Flattery (1877) 2 QBD 410*. The complainant, a 19-year-old lady, visited JF with her mother to discuss a medical condition she was experiencing. JF suggested surgery was necessary. JF engaged in sexual activity with the complainant while pretending to perform surgery. JF was subject to legal action by the crown for rape under Statute 13 Edw. 1, c. 34. The contentious issue in this case was, whether agreeing to engage in sexual activity with JF constitutes consent. The court thought that, the complainant had only agreed to JF's approaches because she thought he was helping her with her epileptic seizures. The court continued that, in cases where the consent was fraudulently obtained, submission did not constitute consent under the law, and that, the plaintiff did not give JF her consent to have sexual relations with her; merely to treat her medically. The complainant agreed to the sexual intercourse under false pretence, the court found, and it was consequently illegal. Judge Mellor cited the case of *R v. Case 19 L. J. (Mag. C.) 174* in support of his argument that "she consented to one thing, he did another that was significantly different, on which she had been precluded by his dishonesty from exercising her judgement and will" (paragraph 414). Mellor cited Statute 13 Edw. 1, c. 34, which defined rape as a sexual activity not "assented [to] before nor after." Mellor came to the conclusion that submitting may be viewed as permission, but not when it was simply provided for another action or item and not for sexual activity. The conviction was upheld, and the appeal was denied.

Ghana’s criminal law also creates the offence of marital rape. Until 2007, Ghanaian law presumed consent to sexual intercourse in marriage at any time. Therefore, a man could not be guilty/accused of rape of his wife. The old section 42(g) of Act 29 read as follows:

“a person may revoke any consent which he has given to the use of force against him, and his consent when so revoked shall have no effect for justifying force; save that the consent given by husband or wife at marriage, for the purposes of marriage, cannot be revoked until the parties are divorced or separated by a judgment or decree of a competent Court” (p. 41).

However, following the promulgation of the Domestic Violence Act, 2007 (Act 732), an amendment was made to Act 29 to create the offence of marital rape. Consequently, section 42(g) of Act 29 revoked the presumed consent between husband and wife during marriage by deleting the part of section 42(g) that states that “save that the consent given by husband or wife at marriage, for the purposes of marriage, cannot be revoked until the parties are divorced or separated by a judgment or decree of a competent Court” (p. 41). The reason for this deletion, according to the memorandum accompanying the Amendment Bill to Parliament was that, the saving provision is unconstitutional (Archampong, 2010). Thus, since 21^st^ February 2007 when Act 732 was passed by the Parliament of Ghana, the criminalisation of rape within marriage took effect in Ghana (Parliament of Ghana, 2007). In furtherance, it therefore follows that since the passage of Act 732, a husband who has sexual intercourse with his wife without her consent, or a husband who continued to have sexual intercourse with his wife who revoked her consent would be guilty of rape committed on his wife. Interestingly however, for a decade of its operation (2007-2017), no case of marital rape has been recorded by the Domestic Violence and Victim Support Unit (DOVVSU) of the Ghana Police Service (Adodo-Samani, 2017). This may not, however, be an indication that, there has not been any such case(s) in our communities. Rather, it may be an indication of the unwillingness of married women to report their husbands to the police. The reasons for this might be the obvious consequence(s) of that report on their marriage, family and social relationships, and children. This contributes to the underreporting of rape cases in Ghana (Office of the High Commissioner for Human Rights, 2020).

Also, as a highly patriarchal society that celebrates male chauvinism (either directly or indirectly) and sometimes sees women (wives) as weaker vessels or even properties of men (their husbands), with time, these women come to psychologically accept these degrading sociocultural beliefs and engage in self-fulfilling prophecies. Therefore, even when offended, in this case, raped by their husbands, they would not report such incidents. In effect, marital rape becomes criminal on paper, but normalised in practice.

## Proof of Rape in Ghana

To prove penetration/carnal knowledge in a rape case, the courts rely on police forensic medical examination reports, that is, a medical report on a police medical form (CID From 98A), signed by a licensed medical practitioner who examines the victim, testifies to the report and is available to be cross-examined on the said report. According to Sharma (2022), a forensic medical examination could be performed on both the accused person and the victim/complainant. Sharma (2022) further noted that, particularly when it comes to establishing the penetrating act's connection to the accused person and proving it, medical evidence from both the victim and the accused is essential for corroboration. According to Patterson et al. (2020), sexual assault forensic examiners (SAFEs) have a complicated job that includes collecting medical forensic evidence and delivering medical care. Generally speaking, SAFEs in this job are guided by two orientations, namely, patient-centered orientation and prosecutorial-centered orientation. Positive emotional results have been associated with a patient-centered orientation/perspective, which stresses listening to emotional needs, providing options, and respecting survivors' decisions (Heyn et al., 2023). A prosecutorial-centered perspective/orientation places a focus on gathering evidence and has been linked to offering less all-inclusive services. The Ghanaian situation at the moment may be described as being more prosecutorial-oriented than patiently-oriented (Patterson et al., 2020).

The procedure usually is that, a female who accuses a male of rape would make a report to the police. The police would then, issue the said alleged victim a police medical form (popularly known as CID Form 98A) to visit any medical facility for examination. The said victim of the rape would then be examined by a licensed medical practitioner, after which the medical practitioner would complete the form and hand it over to the victim, to be returned to the police station/officer where/with whom the complaint had been lodged. This police medical report, would establish a number of facts, becoming proof of carnal knowledge. This report, would first establish whether the victim has indeed been carnally known or not. It would further establish whether, if indeed the victim has been carnally known, it was the accused person who carnally knew her or not (Sharma, 2022). It would further establish whether the victim has been bruised/injured as a result of the rape or not. To establish this, things such as pubic hair/semen deposits of the perpetrator in the vagina of the victim among others are examined by the medical practitioner. This would further require that samples of these items/objects are taken from the accused and compared with those retrieved from the vagina of the victim and analysed by the licensed medical practitioner. Where the medical practitioner’s report makes a positive/confirmatory finding of carnal knowledge of the victim, carnal knowledge stands proved. On an appropriate date during the trial, the medical officer then presents himself/herself at a designated court where the trial of the accused person is ongoing, tenders in evidence the report, and is cross-examined by the accused person or his lawyer on the report.

To prove the age of the victim, the prosecution/police would need to produce the birth certificate of the victim or any other document by which the victim’s age could be established. These documents could include a baptismal certificate, passport, national identity card, driver’s license, and national health insurance card among others. Any document by which the date of birth and for that matter the age of the victim could be ascertained would suffice in this regard.

Consent is the final element that has to be proven by the prosecution in a case of rape. Proof of consent in rape cases is the most contentious and often the only contentious element. This is due to the fact that, consent is a complete defence to rape. This means that, once an accused person in a rape case is able to prove that the victim consented to the sexual intercourse, the case ends and the accused is set free. Therefore, it makes sense that consent is the most contentious issue/element in rape cases. It is also the case that, whereas it is easy to obtain evidence of carnal knowledge, the gender and the age of the victim of rape, the reverse is true of consent. To prove consent therefore, the prosecution must establish that the victim did not voluntarily agree to sexual intercourse with the accused person. Or in the alternative, the prosecution must prove that, even though the victim agreed (consented) to sexual intercourse with the accused, the victim withdrew that consent in the course of the intercourse or that, such consent was wrongfully procured. In practice, proof of such lack of consent would be evidence of injury/bruises/bodily hurt on the victim as a result of struggling with the accused, torn clothes of the victim among others, or even a video recording of the sexual encounter between the victim and the accused person. These pieces of evidence, it must be emphasised, only raise a presumption of lack of consent. The court would thus probe further to establish whether there has been consent or lack of it. Commenting on the element of consent in the US federal law on rape, the United States Department of Justice Archives (2012) stated that, the requirement of consent also covers situations in which the victim is temporarily or permanently incapable of consenting due to physical or mental disability. Additionally, the revised definition acknowledges that drug or alcohol use can render a victim incapable of giving consent and that many rapes are made possible by these substances. Similarly, a victim's age can render them incapable of giving consent. The victim's capacity to provide consent must be assessed in line with each State's specific laws.

## Challenges With Adjudicating Rape Cases in Ghana

Adjudicating rape cases in Ghana comes with numerous challenges, as may be the case in other LMIC of the world. Some of these challenges, in some cases, negatively affect the prosecution of the case to the extent, that the victim withdraws from the case, leading to the case being dismissed or the accused being acquitted and discharged instead of being convicted and sentenced to prison. Statistics from the Ghana Police Service suggest that most rape [and defilement] cases are withdrawn for settlement, which is against the law (Akonor & Okorley, 2021). According to Akonor and Okorley (2021), who gathered information from the Ghana Police Service, Cape Coast, between June 2015 and June 2016 nearly 20 (55.5%) of the 36 rape/defilement cases brought to the unit were withdrawn for settlement outside of court. Victims of rape may also experience additional social and legal issues. Though these authors fell short of mentioning what these legal and social issues are, it is the view of the author of the current article that, the victims of rape are sometimes pressured by their families, society in general and in some cases, religious leaders to withdraw the case for settlement. It has been reported that, a significant barrier to justice in sexual violence cases is social pressure to resolve cases outside of court (de Carvalho & Schia, 2011; United Nations High Commissioner for Human Rights, 2016; Toby, 2021). Other factors identified as responsible for the settlement of rape cases out-of-court include poverty, lax laws, the inefficiency of the legal system, the vulnerability of the relatives of the survivors, and pressure from the family of the criminals. These are just a few of the causes that force survivors and their families to settle outside of court (Toby, 2021).

In some cases, the victims are typically advised by the police to choose financial settlement and accept the pittance being offered by the offender because the courts will simply imprison the accused/offender and will not award any damages to the victim (Akonor & Okorley, 2021). Experience suggests that, in other instances, it is a mere apology (Ghana Statistical Service, 2016). Thus, the police, who are to assist the victim secure justice for the violation of her body and human rights that she has suffered, rather become the source of pressure on the victim to withdraw the case or settle the offence, which settlement is against the law. Where the victim refuses the suggestion of the police, the police sometimes frustrate the victim and her case, in some cases, with delay tactics to the point where the victim becomes disinterested. It is the case therefore that, while the victim has to deal with the psychological trauma she has to endure as a result of the violation of her body and human rights, she also has to endure this social pressure from all angles. The result is that, the victim becomes a “double victim” and disinterested in pursuing the case, leading to its withdrawal or settlement out-of-court.

A major challenge suffered by rape victims in Ghana, is therefore, the poor support the victims of rape sometimes receive from the state/police. Victims of rape in Ghana receive different forms of poor support from the State throughout the process in many respects. One form of poor support victims of rape in Ghana receive is poor support in the forensic medical examination process. When a victim of rape reports the incident to the police, the victim is issued a police medical form to go to any health facility (hospital) for medical examination. Ghana’s media landscape is replete with reports of many victims of rape who cannot afford to pay for their medical examination, thus bringing their cases to naught. According to Ayamga (2020), before the police may investigate their complaint, a rape victim must pay between GHC300 and GHC800 for a medical examination. As paltry as these sums may seem (about 25-35 USD), some victims of rape in Ghana cannot afford due to the high poverty levels. Alluding to this fact, the Ghana Legal Aid Commission's Northern Regional Coordinator, Awudu Issah Mahmudu, emphasised the urgent necessity for the government and the National Health Insurance Authority to make medical examination for rape and defilement victims free of charge. He added that, it is a major injustice and significant disservice to the victims when many victims of physical assault, particularly rape and defilement, are unable to obtain the necessary medical evaluation that is required by the court in order to determine the case's ultimate resolution (Legal Resources Centre, 2023). He encouraged Ghanaians to join the fight to persuade the government and the NHIA to make access to such services in health facilities free for everyone who is a victim in such circumstances, while acknowledging that a medical report is essential to determining the veracity or otherwise of any assault case and must be encouraged. From the above, it can be deduced that medical reports are extremely essential to the determination of rape cases in Ghana and, due to high poverty levels and poor social welfare and support system by the State, some victims of rape are left to suffer in silence.

To investigate help-seeking behaviours, Apatinga et al. (2021) conducted 15 in-depth interviews with married or cohabiting Ghanaian women who had experienced sexual abuse. Financial challenges, a lack of social support, and stigma were just a few of the obstacles that participants mentioned as preventing them from seeking help.

The second challenge faced by victims of rape in Ghana that affect the adjudication of rape cases is the stigmatisation and public ridicule such victims are often exposed to, making them become disinterested in the case, even after mustering the courage to report. Rape victims in Ghana are often ridiculed and stigmatised (Apatinga & Tenkorang, 2022; Brew & Nukpezah, 2022). In some cases, the victims are rather blamed for dressing provocatively in ways that attract/push/lure men into raping them. According to Brew and Nukpezah (2022), reports of rape are frequently ignored and almost never followed through to logical conclusions. In many cases, efforts to prevent rape are restricted to warning girls to take precautions to avoid being raped. Victims who come forward are either blamed or stigmatised. Cannon et al. (2020) also identified stigma and structural barriers, such as cost of medical supplies and lack of privacy within the healthcare facilities as barriers to seeking help by victims of sexual violence in Ghana.

These behaviours, in turn, make the victims become double victims. While suffering from and trying to heal from the traumatic experience of the rape, these victims also had to deal with the public ridicule and stigmatisation they are subjected to. Coupled with the highly patriarchal nature of the Ghanaian society, the end result is that, those who cannot withstand these public pressures and condemnation, simply become disinterested in pursuing the case, even if they have already reported the incident to the police, or, in the alternative, not report the incident at all.

For example, in an alleged rape case in 2015 involving a popular ace broadcaster, Kwesi Kyei Darkwah (KKD) as the accused person and Ewuraffe Orleans Thompson, the victim, State Prosecutor Malike Woanya, told the court that, Orleans Thompson, the complainant, was a virgin who was violently raped by KKD. Prosecutor Woanya continued that a medical report showed Orleans Thompson was bleeding immediately after being raped by KKD in a bathroom at the African Regent Hotel. She noted that, according to the investigation, the 19-year-old had never engaged in sexual activity before. The prosecutor also informed the court that there is enough evidence for her office to bring charges against the well-known broadcaster. Later discussions in the media of the case, the condemnation of the victim, with some even going to the extent of accusing the victim of framing up the offence against the popular ace broadcaster, pushed the victim into withdrawing the case, indicating her intention neither to continue the case nor cooperate with the police, should the police decide to continue the case against her wishes (Daily Graphic, 2015). In a study by Aryee (2013), victims of rape reported of “being embarrassed and stigmatised by society if they became pregnant” (p. 13).

It is to be noted that, the issue of stigmatisation of rape victims is not unique to Ghana but common to other cultures, even Western societies (Jewkes et al., 2022; Khanal, 2022; Schmitt et al., 2021). For example, Khanal (2022) interviewed 10 rape victims who were being cared for by an NGO as part of a study to evaluate the status of rape victims, attitudes against rape, rape myths, and social stigmatisation among respondent groups: victims and community members in Nepal. The majority of the victims in this study were raped while they were teenagers, and 20% of them were victims again, but none of them reported the crimes out of concern for their families' reputations. All the victims were forced to live apart from their family, either permanently or temporarily. In addition, the study indicated that 90% of the victims experienced stigma, with both group victims and community members claiming sexual fulfilment as one of the primary motives of rape.

It is however the case that, in Ghana, the case is pronounced partly because of the patriarchal nature of the society. Also, being a highly moralistic society, coupled with the severe punishment rape attracts, one would not have expected heinous crimes such as rape to be prevalent since society finds such crimes reprehensible (Okafor-Udah, 2015; Reshma et al., 2022).

Closely linked to the above is the lack of social and psychological support for victims of rape in Ghana. Rape is one of the most traumatic experiences a woman can have, which can have long-term effects like post-traumatic stress disorder (PTSD), suicidal thoughts, depression, and other health consequences (Nautiyal et al., 2017; Reshma et al., 2022). It has been discovered that, compared to women who were raped by strangers, those who were familiar to them, such as close relatives, had higher levels of depression. Alcoholism disorders, eating disorders, anxiety, depression, despair, self-harm, and suicidality are other mental health side effects (WHO, 2013). Rape has also been found to have indirect connections to bad long-term health outcomes; for instance, using lifetime PTSD as a proxy, PTSD is linked to a higher risk of hypertension, cardiovascular disease, and digestive issues (Brown et al., 2019). Furthermore, young unmarried women who fall pregnant are typically dismissed from school in Ghana, regardless of the circumstances surrounding their pregnancy. They might also be left on their own by their families after being abandoned. Some are left on their own to cover the rape victim's medical costs (Ardayfio-Schandorf, 2005; Ark Foundation, 2011; Aryee, 2013; Kuenyehia, 1998; UNFPA, 2012). Fear that rape could result in pregnancy could again enhance depression in the same way as HIV-related anxiety can.

Despite these findings, rape victims in Ghana receive no psychological or social support. Rather, they are taken through long torturous court proceedings that sometimes take years to be completed. They are exposed to their perpetrators without first psychologically orienting and strengthening them to face them. At the end of the trial, the accused person, if found guilty, is sentenced to a term of imprisonment between 5 and 25 years, in hard labour. However, the victim receives absolutely no form of support, compensation or “treatment” for the harm caused to her. The victim is accordingly, left to suffer in silence. Meanwhile, in addition to having detrimental effects on physical and mental health, Brown et al. (2019) found that rape and sexual assault have long-lasting social and economic implications that hinder people's ability to work, raise a family, and participate in community life. In addition to the negative effects on one's physical and mental health, rape and sexual assault have a major negative societal and economic impact due to missed productivity, the cost of the police and the criminal justice system, and other factors. Therefore, while the lack of psychological support for victims of rape becomes a disincentive to report and/or pursue rape cases in Ghana, it also has long-term effects on the physical and mental health/wellbeing of the victim, while at the same time, affecting future economic life of the victim and the nation as whole.

Similarly, the offender also receives no form of evidence-based practice (EBP) rehabilitation for his crime. In reality, there is almost no offender treatment during the period of incarceration, apart from general faith-based approaches aimed at prison inmates’ reformation. In a study by Darkwa (2018) on factors contributing to recidivism among inmates in the Nsawam Medium Security Prison in Ghana, a recidivist revealed that, “we are too many with different charges but share the same cell. As a result, some copy from others and go out and practice it. A person may first come here with a traffic charge and the next time he comes with robbery or rape charge. Some learn how to steal, rape, and smoke and they meet outside after discharge, form a gang and commit another crime” (p. 48). According to research by Afari, Osei, and Adu-Agyem (2015) on recidivism at the Kumasi Central Prison, specifically looking at the guidance and counselling services available at the prison for the reformation of prisoners, it was found that prisons in Ghana lack qualified and sufficient professional counsellors. This research further observed that, as a result of overcrowding in the prisons, it is extremely challenging for counsellors to address inmates' needs for counselling. Similarly, in a separate study, Harrison et al. (2020) observed that, recidivism among individuals/prisoners who perpetrate sexual offences can be significantly reduced using cognitive-behavioural therapy models. A study in 9 prisons in South Korea on the effectiveness of treatment programmes for individuals who have been convicted for sexual offences revealed that high-risk offenders may continue to experience larger levels of issues with emotional loneliness, anger management, and impulsivity even after receiving sexual offender therapy (Yoon & Knight, 2013). This study highlights the need for treatment for individuals who perpetrate sexual offences, while emphasising that, such treatment programmes must be long-termed, even spanning periods after incarceration.

The conclusion from these findings is that, prisoners in Ghana, in this case, those who have been convicted for rape, have little chance of reformation, due to the lack of/inadequacy of reformative services such as guidance and counselling in the prisons for them. Like one recidivist observed in the study by Darkwa (2018), they return after serving their sentences, hardened, and learning more criminal activities than what took them to prison in the first place.

Yet, another challenge with the adjudication of rape cases in Ghana is the mode of trial of such cases. According to the Supreme Court of Ghana in the case of *Richard Banousin v. The Republic [2014] GHASC 10*, every rape case must be tried (adjudicated upon) by a judge and jury since rape is an indictable offence, that is, a first-degree felony. According to Part IV of the Criminal (Procedure) Act, 1960 (Act 30), such a trial must commence with a committal process (preliminary/mini-trial) fraught with several technicalities and delays before the accused is finally committed to stand trial in the High Court. Being a first-degree felony, the case must be tried by a judge and jury. Jury trials have been found to be fraught with many challenges (DeCamp & DeCamp, 2020; Duff, 2001; Flanagan, 2015; Zeisel & Diamond, 1978), sometimes, leading to the accused being acquitted and discharged instead of being convicted and sentenced (Salerno et al., 2023).

Closely linked to the mode of trial as a challenge in the adjudication of rape cases in Ghana is the long duration such cases take. On average, trial of an accused person on charges of rape could take at least a year or even more. By the time the jury is empanelled and the full trial commences, more than six months have passed. The trial process itself could take not less than another six months. A study by Anzagra et al. (2013) in the Upper East and West Regions of Ghana on the average time it takes for judgement to be rendered on a case after it has been reported or after a complaint has been made found that, the average time to justice delivery for cases in the circuit court was 229% less than that for cases in the district court. When additional factors were taken into account, the average time to justice delivery in criminal cases was 52% longer than in civil cases. However, the average time to administer justice increased by 21% for every additional hearing. According to these authors, the increased frequency of following hearings was linked to complex cases like rape, defilement, murder, among others (Anzagra et al., 2013).

## Conclusion and Recommendations

This paper assesses the offence of rape in Ghana from the perspectives of the law, the victim, and the offender. It commences by examining what rape is, the Ghanaian law on rape and key elements in the definition of rape in Ghana, supported by case law. The paper then discusses the challenges associated with adjudicating rape cases in Ghana. Key among these challenges are the financial constraints the victim of rape face in undergoing a medical examination to ascertain whether there has been a penetration, the ridicule and stigmatisation victims of rape are subjected to by the public, the lack of psychological and social support for victims, and the inadequacy or lack of reformative services in the prisons for the reformation of rape prisoners.

The first recommendation this paper makes is the amendment of section 98 of Act 29 on the definition of rape. Like the US, UK, Kenya and South Africa, it is high time for Ghana to amend its criminal law on rape, to make it gender-neutral instead of gender-specific. It does not accord with science and modernity to still maintain that, a man cannot be raped.

Secondly, medical examination for rape victims, a key evidential element in establishing rape, must be made free for all victims. The State must pay for such an examination, instead of the current practice of having the victim bear the cost of the medical examination. This will go a long way to encourage more victims of rape to report their ordeals to the police, and pursue the case to its fullest conclusion. In jurisdictions such as the US (Zweig et al., 2014) and India (Math & Harbishettar, 2019), the State pays for forensic medical examination for victims of sexual violence. In the absence of such state-funded forensic medical examinations, public health insurance services pay for such examinations. Ghana must learn from the example of these countries and either directly cover the cost of medical examination of rape victims or direct the NHIA to cover such costs.

Furthermore, there must be public education, awareness creation, and other interventions to reduce stigma and discrimination against rape victims in Ghana. After having experienced such a traumatic and lifetime-scarring ordeal, the least a victim of rape would expect from society and deserves is social and psychological support, and not stigmatisation. According to Jewkes et al. (2022), by addressing concerns of guilt and stigma as part of basic treatment for rape survivors, gender-empowering psychological first aid may be able to lessen the impact of rape stigma. Harrison et al. (2020) emphasised that sexual offenders face stigma from a variety of groups, including law enforcement, the media, the community, and non-sexual offenders. They are also continuously denigrated in general discourse, research focus, and article titles. To deal with this issue, Murray et al. (2018) suggested that, cognitive-behavioural therapies may be a useful tool for reducing stigma for sexual assault survivors. They further found that, experiences with or perceptions of stigma did not impair the therapeutic effects of group psychotherapy on survivors' mental health. If the public ridicule and stigmatisation continue, Ghana would continue to experience underreporting of rape cases or even when reported, the victims would later withdraw from the case. This, clearly, would not augur well for the image and international reputation of the country. Such cases must also be dispensed with early, in order to prevent the victims from becoming disinterested as a result of delays.

It is also recommended that, the government through the Attorney-General must issue a fiat, directing rape cases to be tried summarily instead of being tried on indictment, as has been done for robbery cases. This would resolve two challenges confronting the adjudication of rape cases in Ghana. First, it would fast-track/speed up the trial of rape cases since the case would be tried only by a judge and thus eliminate issues of peremptory challenges among others (Flanagan, 2015). Second, it would also eliminate situations where accused persons are acquitted and discharged instead of being convicted by the jury.

Next, medical and psychological treatment for victims of rape must be made an integral part of the criminal justice system as far as the adjudication of rape cases in Ghana is concerned. This treatment must be holistic, encompassing EBP, and paid for either by the State or the offender. This treatment should be targeted psychotherapeutic treatment that is individualized for the victim. For the treatment of individuals who have experienced sexual assault and abuse, Cowan et al. (2020) identified psychodynamic psychotherapy, trauma-focused cognitive behavioural therapy (TF-CBT), and eye movement desensitisation and reprocessing therapy (EMDR).

Also, as suggested by Patterson et al. (2020) the individual who perpetrates sex crime must also be treated, just like the victim. Research reveals that offense-focused psychological treatment for sexual offenders may be somewhat helpful in lowering rates of both sexual and general reoffending, (Tyler et al., 2021). These treatments are frequently psychological and given in a variety of places, including the community, forensic mental health facilities, and prison settings. Thus, when convicted and imprisoned, the perpetrators of sexual offences must be taken through thorough programmes and services aimed at reforming them. Incarcerating the perpetrators of rape is not enough, if the incarceration is not aimed at reformation. In this regard, the government must resource the Ghana Prisons Service, provide the Prisons Service with more funding and other facilities so that they fully discharge their mandate of reforming prisoners, in this case, rape prisoners. Prior to these recommended interventions/treatments, Lussier et al. (2023) also suggest the evaluation of a sexual offender's mental health (such as personality disorder and psychopathy), psychological, sexual, and social functioning (such as cognitive distortions supporting rape and sexual assault), as well as their individual criminal history (such as prior conviction for any crime), past sexual offending (such as prior conviction for a sex crime), and victim characteristics (such as male and under 12 years old). According to these authors (Lussier et al., 2023), these factors are among the most robust factors predictive of sex offending found in general samples of offenders (samples that are not limited to offenders who have committed sexual offenses). Such an assessment would therefore, go a long way in significantly reducing sex offender recidivism in Ghana, a phenomenon found by Afari et al. (2015) and Darkwa (2018) to be high in Ghana’s prisons.

Finally, perpetrators of rape must be made to compensate their victims. They must be made to compensate their victims with an amount of money as reparation. As exists in countries such as South Africa and the UK, victims of rape in Ghana must be compensated by the perpetrator (University of Oxford, 2016). Where the perpetrator is a man of no means and incapable of compensating the victim, the State must step in to compensate the victim.
